# Determination of circuit-specific morphological adaptations in ventral tegmental area dopamine neurons by chronic morphine

**DOI:** 10.1186/s13041-019-0435-6

**Published:** 2019-02-08

**Authors:** Sarah C. Simmons, Katie Wheeler, Michelle S. Mazei-Robison

**Affiliations:** Neuroscience Program and Department of Physiology, Michigan State University, 567 Wilson Road, BPS 3182, East Lansing, MI 48824 USA

**Keywords:** Dopamine, Morphine, Morphology, Opiate, Ventral tegmental area

## Abstract

Chronic opiate exposure induces neuroadaptations in the mesocorticolimbic system including ventral tegmental area (VTA) dopamine (DA) neurons, whose soma size is decreased following opiate exposure. Yet it is now well documented that VTA DA neurons are heterogeneous, with notable differences between VTA DA neurons based on their projection target. Therefore, we sought to determine whether chronic morphine induced similar changes in the morphology of VTA DA neurons that project to the nucleus accumbens (NAc) and prefrontal cortex (PFC). We utilized Cre-dependent retrograde viral vectors in DA Cre driver lines to label VTA DA neurons that projected to NAc and PFC and assessed neuronal soma size. Consistent with previous data, the soma size of VTA DA neurons that projected to the NAc medial shell was decreased following morphine exposure. However, soma size of VTA DA neurons that projected to the NAc core was unaltered by morphine. Interestingly, morphology of PFC-projecting VTA DA neurons was also altered by morphine, but in this case soma size was increased compared to sham controls. Differences in basal soma size were also noted, suggesting stable differences in projection-specific morphology in addition to drug-induced changes. Together, these data suggest morphine-induced changes in VTA DA morphology occur within distinct VTA DA populations and that study of opiate-induced structural plasticity of individual VTA DA subcircuits may be critical for understanding addiction-related behavior.

**Electronic supplementary material:**

The online version of this article (10.1186/s13041-019-0435-6) contains supplementary material, which is available to authorized users.

## Introduction

There is increasing concern in both the scientific and public domains regarding the United States opioid epidemic, highlighted by a 500% increase in opioid overdose deaths from 1999 to 2016 [5]. Long-term use of opioids (either in prescription or illicit forms) is frequently concurrent with escalating intake of drug and increased risk for physical drug dependence and addiction [4, 14]. It is well established that the mesocorticolimbic circuitry is integral in the addictive properties of several drugs of abuse, including opiates, and that drug-induced changes to the mesocorticolimbic circuitry are central to the development of drug abuse [12, 21, 26].

Long-term use of opiates, such as morphine or heroin, can have lasting effects within the mesocorticolimbic circuitry, particularly the ventral tegmental area (VTA) dopamine (DA) neurons, which are essential for the processing of reward. For example, following chronic morphine administration VTA DA neurons exhibit increased spontaneous and burst firing rates [23, 33] and a unique morphological adaptation, specifically an ~ 20–25% reduction in soma size [9, 33, 41, 43]. Given that this reduction can be blocked with opiate antagonists such as naltrexone [43] and that it does not occur following chronic administration of other commonly abused drugs such as cocaine, ethanol, or nicotine [32] suggests that this structural plasticity may be dependent on opioid signaling. Decreased VTA DA soma size has also been observed in post-mortem samples from heroin addicts, demonstrating translational relevance [33]. Importantly, this opiate-induced decrease in soma size is also correlated with behavioral changes such as reward tolerance; higher doses of morphine are required to elicit a conditioned place preference concomitant with decreased soma size [41]. Together, these data suggest that chronic morphine elicits distinct morphological and electrophysiological responses in VTA DA neurons, and that these changes are concurrent with changes in the behavioral state of the subject.

While evidence supports opiate-induced changes in VTA DA morphology, whether these changes are limited to subpopulations of VTA DA neurons remains unclear. These distinctions may be critical as current work highlights that the activity of subpopulations of VTA DA neurons drive distinct behavioral states. For instance, activity of nucleus accumbens (NAc)-projecting VTA DA neurons has long been associated with rewarding aspects of stimuli [42, 47], but more recently increased activity of these neurons has been shown to promote susceptibility to chronic social defeat stress, a depressive-like phenotype [7]. In contrast, activity of prefrontal cortex (PFC)-projecting VTA DA neurons appears to regulate processing of aversive stimuli such as formalin paw injection [25]. Subpopulations of DA neurons also display different basal electrophysiological properties. For example, VTA DA neurons that project to the PFC, medial NAc shell (m.shell), and NAc core have a small hyperpolarization-activated current (I_h_) and higher maximal firing frequencies whereas neurons projecting to the lateral NAc shell (l.shell) have a high I_h_ and lower maximal firing frequency, similar to DA neurons in the substantia nigra (SN) that project to the dorsal striatum [24, 25]. This distinction is important due to the historical use of high I_h_ to identify VTA DA neurons, which may not only bias against recording from subsets of VTA DA neurons with low I_h_, but also includes a subset of TH-negative neurons that have a high I_h_ [31], complicating interpretation of the role of DA neurons in studies utilizing this criterion.

In spite of the growing literature describing specific circuits underlying reward and aversion, there is a surprising lack of information on circuit-specific adaptations to opiates in the mesocorticolimbic system. Thus, we sought to determine whether chronic morphine induces morphology changes within specific subsets of VTA DA neurons. NAc- and PFC-projecting VTA DA neurons were identified using Cre-dependent retrograde viral tracers in DA-Cre driver lines and basal and morphine-induced changes in morphology were assessed. Together, the results of this study further establish that VTA DA neurons are not homogeneous, as they show diverse basal morphology. Importantly, we reveal that chronic morphine induces opposing effects in discrete physiologically relevant VTA DA populations.

## Materials and methods

### Animals and stereotaxic surgery

For all experiments, we used adult (> 8 weeks) male and female tyrosine hydroxylase (TH)-Cre (Jackson, Cat# 008601, RRID:ISMR_JAX:008601) and Slc6a3 (dopamine transporter, DAT)-Cre (Jackson, Cat# 006660, RRID:ISMR_JAX:006660) mice. All experimental animals had ad libitum access to standard chow and water and were kept on a 12 h light-dark cycle. All experiments were approved by the Michigan State University Institutional Animal Use and Care (IACUC) committee and adhered to the guidelines set in the Guide for the Care and Use of Laboratory Animals of the National Institutes of Health. Stereotaxic surgeries were completed following standard protocols [6, 22, 25]. Briefly, mice (8–9 weeks) were anesthetized using ketamine (100 mg/kg) and xylazine (10 mg/kg) and viral vectors were bilaterally infused (0.5 μl over 5 min) into the brain projection region of interest (e.g. NAc or PFC). Specifically, retrograde viruses (AAV5-EF1a-DIO-eYFP-WPRE-hGH and AAV5-EF1a-DIO-mCherry, University of Pennsylvania and University of North Carolina Vector Cores, respectively) were targeted to either the NAc (AP: + 1.6, ML: + 1.5, DV: -4.4) or mPFC (AP: + 1.8, ML: + 0.65, DV: -2.0). Mice were then returned to their home cages (with conspecific littermates) for 6–8 weeks to allow for complete retrograde transport and stable fluorescent protein expression.

### Morphine treatment

Six to eight weeks following stereotaxic surgery (14–17 weeks old), mice were subcutaneously implanted with either sham or morphine (25 mg) pellets (generously supplied by the NIDA Drug Supply Program) under isoflurane anesthesia as previously described [16]. Briefly, mice were implanted with single subcutaneous pellets on days 1 and 3 and were then sacrificed on day 5 (Fig. 2b), a validated protocol to produce morphine dependence and changes in VTA DA neuronal activity, morphology, and signaling [16, 23, 33].

Analysis of blood morphine serum concentration was completed at the Michigan State University Mass Spectrometry and Metabolomics Core. A separate cohort of mice (6 male and 8 female) was implanted with morphine pellets according to the procedure above. Trunk blood was collected following decapitation and serum was isolated using silicone-coated BD-Vacutainer tubes (BD-367812) and centrifuged at 1300×g for 5 min at 4 °C. Free-morphine concentration was determined using Acquity TQ-D LS/MS-MS mass spectrometry utilizing an internal standard of Morphine-D3 (Lipomed, M39-FB-1LM) and standard curve generated with morphine sulfate (Cat# M8777, Sigma). Serum proteins were removed with 3:7 dilution into acetonitrile and centrifuged at 1000×g for 3 min at 4 °C and morphine-containing diluent were subsequently diluted in water for analysis. Final free-morphine serum concentrations were normalized to mouse weight (nM/g).

### Immunohistochemistry (IHC)

Mice underwent transcardial perfusions with PBS and 4% paraformaldehyde (pH 7.4) under chloral hydrate anesthesia and brains were removed and post-fixed for 24 h in 4% paraformaldehyde and then placed in 30% sucrose at 4 °C until further processing. Brains were sectioned at 40 μm using a freezing microtome and sections were stored in PBS + 0.01% sodium azide at 4 °C. Immunohistochemistry was completed on sections containing VTA, NAc, and PFC following standard procedures [33]. All incubations and washes were completed at room temperature, PBS was used for washes and sections were blocked in PBS with 3% normal donkey serum (NDS, Jackson ImmunoResearch Laboratories, Cat# 017–000-121, RRID:AB_2337528) and 0.3% Triton X-100. Primary antibodies were prepared in PBS with 3% NDS and 0.3% Tween-20 and sections were incubated in primary antibody overnight at room temperature. The following primary antibodies and dilutions were used: mouse anti-TH (Sigma, Cat# T1299, RRID:AB_477560, 1:5000), rat anti-mCherry (Invitrogen, Cat# M11217, RRID:AB_2536611, 1:20,000), rabbit anti-GFP (Life Tech, Cat# A11122, RRID:AB_221569, 1:18,000), rabbit anti-gamma synuclein (Sncg, Abcam, Cat# 55424, RRID:AB_2193398, 1:2000), rabbit anti-sex determining region Y-box 6 (Sox6, Abcam, Cat# 30455, RRID:AB_1143033, 1:500), and goat anti-orthodenticle homeobox 2 (Otx2, Neuromics, Cat# GT15095, RRID:AB_2157174, 1:200). To determine viral spread in in NAc/PFC tissue of DAT-Cre mice, primary antibody concentrations for rat anti-mCherry and rabbit anti-GFP were increased to 1:10,000 due to weaker terminal signal. All secondary antibodies were from Jackson ImmunoResearch and diluted 1:500 in PBS: anti-Rabbit-488 (Cat# AF-711-545-152, RRID:AB_2313584), anti-Rat-594 (Cat# AF-712-585-153, RRID:AB_2340689), anti-Mouse-CY5 (Cat# AF-115-175-146, RRID:AB_2338713), and anti-Goat-CY5 (Cat# AF-705-175-147, RRID:AB_2340415): sections were incubated in secondary antibodies for 4 h at room temperature. Sections were mounted on standard slides, dehydrated in 70–100% ethanol, and cover-slipped using DPX mounting medium (Electron Microscopy Sciences, Cat# 13512).

### Microscope image acquisition

All slides were coded and experimenters were blind during image acquisition and analysis; samples were not decoded until analysis for all samples was complete. For soma size studies, VTA confocal z-stack images were taken using Olympus Fluoview FV1000 (version 4.2) and digital color camera (Olympus DP72) using a 60x PlanApoN (NA 1.42) oil objective and scan speed of 8.0 μs/pixel, Kalman average 4, step size 0.42 μm (Michigan State University Center for Advanced Microscopy). Following confocal imaging, neurons expressing either eYFP or mCherry (fluorescent protein, FP) were reconstructed in three dimensions and the surface area was measured using image analysis software (Volocity 3D Image Analysis Software, RRID:SCR_002668) as described previously [32, 33, 41]. We observed robust co-localization of TH protein (IHC) and viral-mediated fluorescent labeling with very few TH-negative, FP-positive neurons in the posterior lateral regions of the VTA (paranigral, PN and lateral portion of the PN) consistent with previous studies [44]. However we did note more TH-negative, FP-positive neurons in the posterior midline region (interfascicular nucleus, IF) as previously reported [28]. While all co-labeled neurons within the scan were sampled, only FP labeled neurons that were co-labeled with TH-IHC and showed complete 3D fill were included in the soma size study. For statistical analyses, soma surface areas of 4–38 neurons were averaged for each mouse. The n listed in figure graphics refers to the number of mice per group, which was used for the statistical analyses, and the range of neurons averaged to get a value for each mouse is listed in the figure legend and listed in Additional file 1: Table S1. The quantification of the individual cell soma size for all cells analyzed in the study are listed in Additional file 2: Table S2.

For determination of viral targeting, NAc, PFC, and VTA were imaged using a Nikon 600HL eclipse NiU upright microscope, Lumencof sola light engine, and Photometrics cool SNAP Dyno camera. NAc and PFC were imaged using a 10X/0.3 Plan Fluor DIC LN1 objective and VTA was imaged using a 20X/0.5 Plan Fluor DIC MN2 objective. FITC, TexRed, and CY5 filters were used determine IHC-labeled eYFP, mCherry, and TH (Cy5) expression. Combined viral targeting images were created by overlaying images of the same Bregma region from each animal. Individual images for each region were acquired as follows: NAc medial shell and core (Bregma + 1.10): FITC-400 ms, TxRed-700 ms; PFC (Bregma + 1.78): FITC-400 ms, TxRed-1 s. The final combined images were converted into grey scale and then into a heat map (fire green blue). The final image LUTs were adjusted as follows: NAc medial shell- 500-1000, NAc core- 200-1000, PFC- 500-2000.

For colocalization studies, only neurons expressing Cre-driven mCherry (e.g. DA projection neurons) were assessed for Sncg, Otx2, or Sox6 colocalization within the VTA (PN, L.PN, and PBP) and SNc. Confocal z-stack images were taken using Olympus Fluoview FV1000 (version 4.2) and digital color camera (Olympus DP72) using a 20X and 40X dry objective and scan speed of 8.0 μs/pixel, Kalman average 4, step size 1.8 μm. Counts of mCherry expressing DA neurons positive for Sncg, Otx2, and Sox6 were obtained by an experimenter blind to conditions (*n* = 4/5 mice/group). All colocalization data are represented as the percentage of DA projection cells: (# cells positive for protein marker (e.g. Sncg) and mCherry) / (total # of mCherry-positive cells) × 100.

### Statistical analyses

All statistical analyses were performed using GraphPad software (Prism, version 7). Results from experiments with two experimental groups were analyzed using an unpaired student t-test. Results from studies with 3 or more groups were assessed using one- or two-way ANOVAs followed by Tukey post-hoc tests, when appropriate. *p* values less than 0.05 were considered significant.

## Results

### Characterization of basal soma size of VTA DA projection neurons

**Fig. 1:**
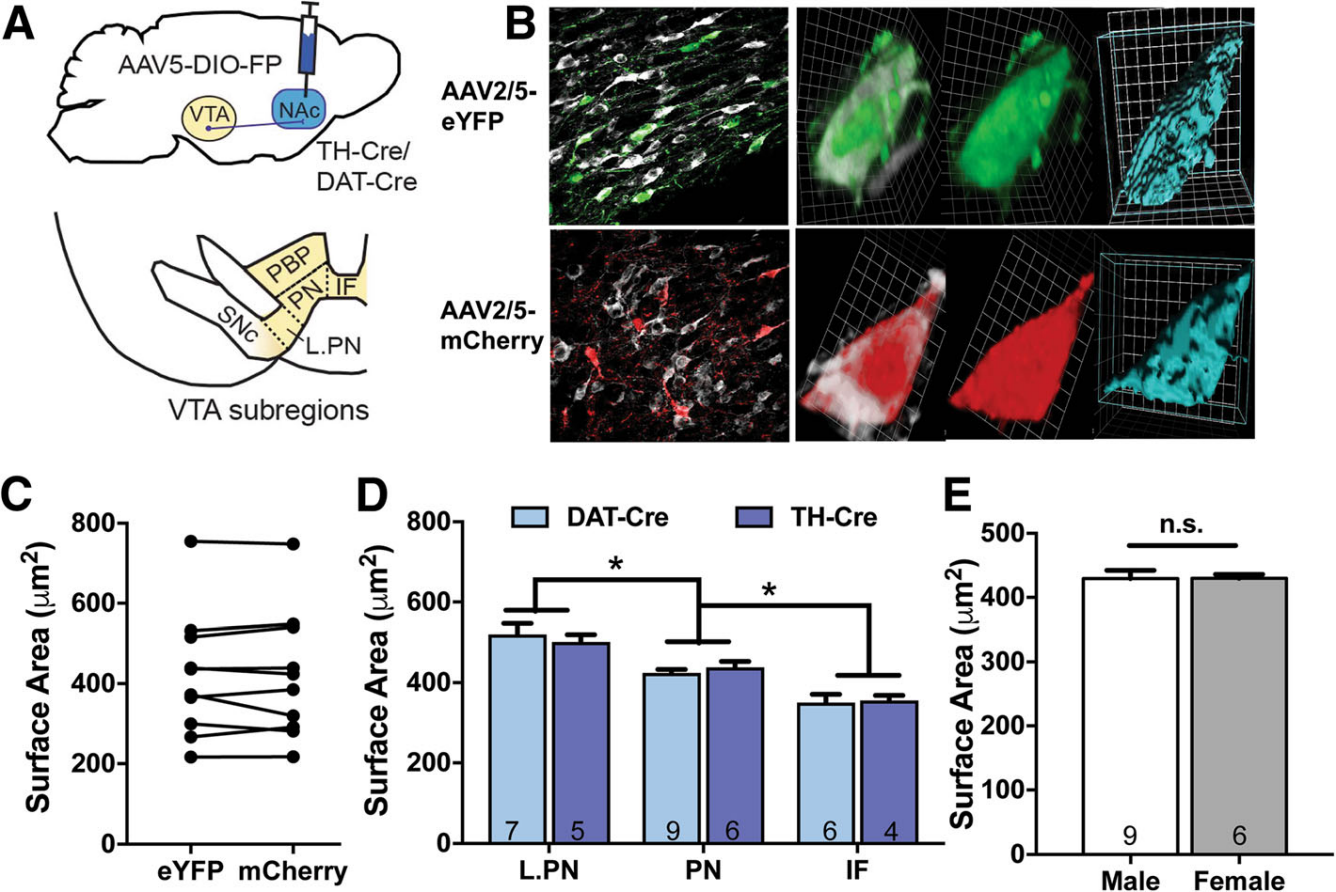
Basal soma size characterization of NAc-projecting VTA DA neurons. **a**. TH-Cre or DAT Cre-mice received bilateral infusions of retrograde AAV5-DIO-eYFP or –mCherry into the NAc and soma size of VTA DA neurons was analyzed in VTA subregions (interfascicular nucleus (IF), parabrachial pigmented nucleus (PBP), paranigral nucleus (PN), lateral portion of paranigral nucleus (L.PN)) **b**. Representative images of VTA DA neurons in TH-Cre mice labeled with AAV-DIO-eYFP (green, top) or –mCherry (red, bottom) showing colocalization with TH (white) and soma reconstruction using Volocity (blue). **c**. VTA DA neurons expressing both fluorophores (FP, eYFP and mCherry) had comparable surface area measurements using either FP construct (*n* = 8 neurons). **d**. Basal VTA DA soma size differed between VTA subregions in TH-Cre and DAT-Cre mice, with no differences observed between DA-driver lines. The number of mice in each group is noted within columns, 4–23 neurons were analyzed per mouse. *denotes significant subregion effects in both TH-Cre and DAT-Cre mice (two-way ANOVA, Tukey’s post-hoc test, *p* < 0.05). **e**. VTA DA soma size (PN subregion) did not differ between male and female mice. The number of mice in each group is noted within columns and 9–38 neurons were analyzed per mouse (student t-test, n.s., *p* > 0.05)

To first establish that both AAV5-DIO-eYFP and AAV5-DIO-mCherry similarly label VTA DA projection neurons, both vectors were stereotaxically injected into the NAc of TH-Cre and DAT-Cre mice (Fig. 1a). Surface area measurements in VTA DA neurons expressing both eYFP and mCherry were assessed to validate that labeling with either fluorophore produced similar results. Indeed, surface area calculations were highly correlated (Pearson r = 0.9883, *p* = 8.2 × 10^− 18^), suggesting comparable cell labeling (Fig. 1b, c). Therefore, in all future studies eYFP and mCherry were counter-balanced across all experimental groups and surface area data were combined. To address any differences in viral-mediated targeting of dopamine populations [28, 45], we compared VTA DA soma size using TH-Cre and DAT-Cre mice across VTA subregions [2, 3, 19]. Using a two-way ANOVA, we determined a significant effect of VTA subregion (F_(2,31)_ = 36.27, *p* = 7.62 × 10^− 9^), but no effect of Cre-driver (F_(1,31)_ = 4.83 × 10^− 6^, *p* = 0.998) and no significant subregion x Cre-driver interaction (F_(2,31)_ = 0.4117, *p* = 0.67). Post-hoc analyses revealed that all subregions analyzed (interfascicular nucleus (IF), paranigral (PN) and lateral portion of the PN (L.PN), see Fig. 1a lower panel) significantly differed from each other (Fig. 1d, Tukey’s multiple comparison test, *padj. < 0.05; DAT-Cre: L.PN vs. PN *p* = 4.99 × 10^− 4^, L.PN vs. IF *p* = 3.33 × 10^− 7^, PN vs IF *p* = 0.0098; TH-Cre: L.PN vs. PN *p* = 0.064, L.PN vs. IF *p* = 8.8 × 10^− 5^, PN vs. IF *p* = 0.020). Soma size increased along the medial-lateral axis such that the smallest VTA DA neurons were located within the medial IF and the largest neurons in the L.PN in both TH-Cre and DAT-Cre mice (Fig. 1d). Since VTA DA neurons in TH-Cre and DAT-Cre mice were similar in size, a single model (TH-Cre) was used in subsequent morphine studies and distinct subregions were analyzed separately in order to avoid any confounds of basal differences in soma size. Finally, because previous soma size data was exclusively from males, we compared the basal soma size of male and female mice. We found no significant difference between the soma size of VTA DA neurons (PN subregion) of male and female DA-Cre mice (Fig. 1e, student t-test, t_(13)_ = 0.007953, p = 0.99) consistent with observations that there are no sex differences in the basal electrophysiological properties, connectome or translatome of VTA DA neurons [10]. Thus, in subsequent experiments we combined data from males and females.

### Morphine-induced changes in soma size are projection-specific

**Fig. 2:**
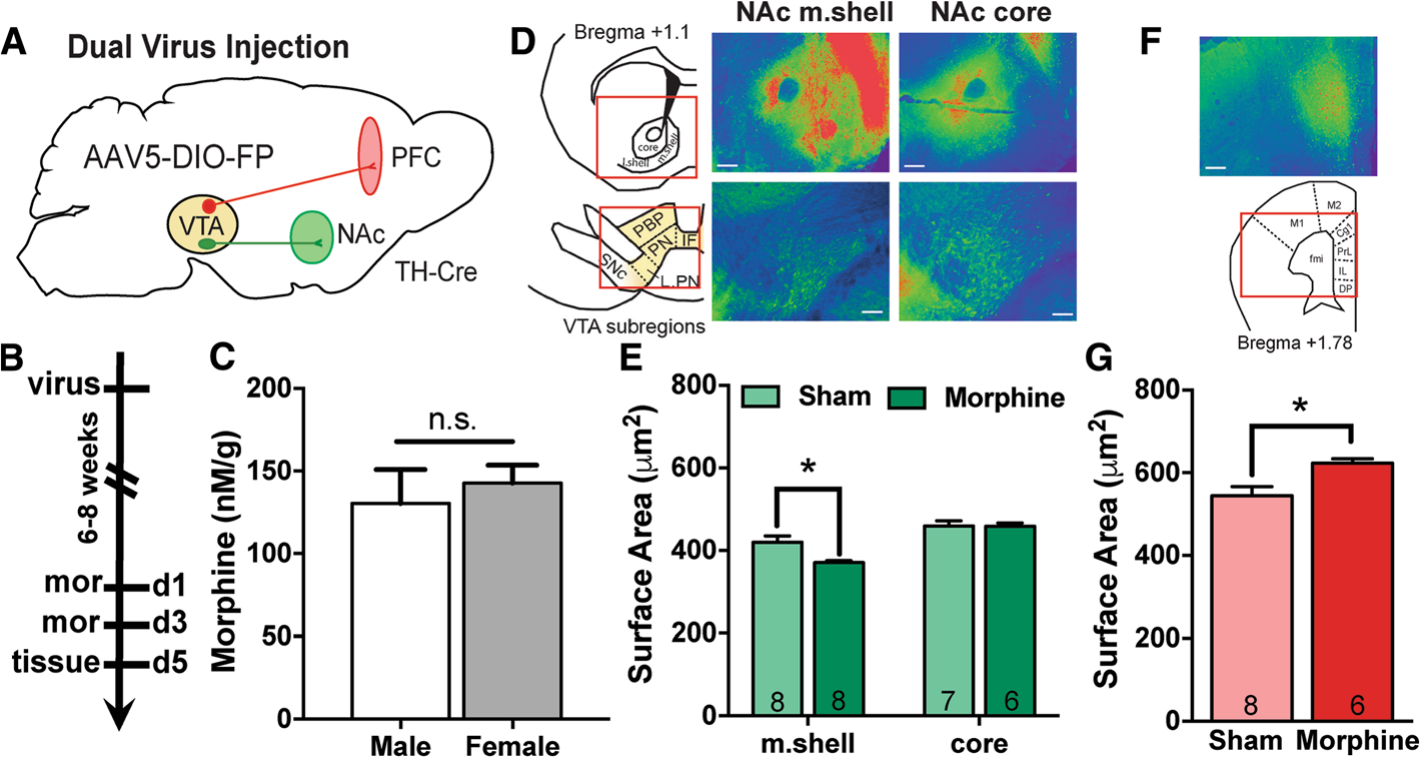
Chronic morphine effects on VTA DA soma size differ based on projection target. **a**. TH-Cre mice received bilateral infusions of retrograde AAV5-DIO-FP into the NAc or PFC and VTA DA neuron soma size was measured following chronic morphine or sham pellet administration. **b**. Timeline of experimental paradigm. Morphine or sham pellets were implanted on days 1 and 3 (d1, d3) and tissue was collected on day 5. **c**. Male and female mice showed similar free morphine concentration in blood serum (student t-test, n.s., p > 0.05) **d**. Heatmaps of combined images used in morphine studies showing distinction in NAc medial shell (m.shell)- and core-projecting VTA DA neurons. Representative images were taken using 10X objective, scale bar = 100 μm. Top images: NAc infusion sites, Bottom images: labeled VTA DA neurons. **e**. Morphine decreases the soma size of NAc m.shell-projecting VTA DA PN neurons with no effect on VTA DA PN neurons that project to the NAc core. Number of mice in each group is noted within columns and 7–26 neurons were analyzed per mouse. *denotes significant effect of morphine on soma size (two-way ANOVA, Tukey’s post-hoc test, p < 0.05). **f**. Heatmap of PFC infusion sites used in morphine studies. Representative images were taken using 10X objective, scale bar = 100 μm. **g**. Soma size of VTA DA neurons that project to the PFC is increased following chronic morphine treatment. Number of mice in each group is noted within columns and 4–12 neurons were analyzed per mouse (student t-test, *p < 0.05)

Chronic morphine decreases the soma size of VTA DA neurons, but it remains unclear whether all DA neurons display this response or if it is limited to subsets within the VTA. In order to determine if morphine-induced effects on morphology occur in a projection-specific manner, the surface area of NAc- and PFC-projecting VTA DA neurons were compared in sham- and chronic morphine-treated TH-Cre mice (Fig. 2a and b). Prior experiments that implanted subcutaneous morphine pellets for chronic morphine exposure exclusively used male rodents [32, 33, 41]. Therefore, we tested the drug administration paradigm in both sexes, and found no significant difference in free morphine (nM/g mouse) in isolated blood serum (Fig. 2c, student t-test t_(12)_ = 0.564, *p* = 0.58). Given this, and the observation that basal soma size is equivalent between the sexes (Fig. 1e), we combined data from male and female mice for morphine soma size measurements. Targeting of the projection site was validated by fluorescent protein (FP) expression; images of injection sites (heat map of florescence) are shown for NAc subregions (Fig. 2d) and PFC (Fig. 2f). Consistent with previous observations [24, 25], injections centered in the NAc m.shell labeled VTA DA neurons predominately in the medial and ventral regions of the VTA (IF, PN, L.PN), while injections centered in the NAc core also labeled VTA DA neurons in PN and L.PN, but in addition labeled more neurons in the PBP, likely due to diffusion into the l.shell (Fig. 2d). PFC injections were centered in the Cg1/Prl PFC and labeled VTA DA cells were predominately in the PBP, with sparse labeling in the PN. To control for basal differences between subregions (Fig. 1c), NAc-projecting VTA DA neurons (m.shell and core) were analyzed specifically within the PN region and PFC-projecting VTA DA neurons (within the PBP) were analyzed separately.

We found a significant main effect of drug (F_(1,25)_ = 4.872, *p* = 0.037), a main effect of projection (F_(1,25)_ = 30.93, *p* = 8.78 × 10^− 6^), and a significant drug x projection interaction (F_(1,25)_ = 4.382, *p* = 0.047) in VTA DA neurons projecting to the NAc m.shell and core (Fig. 2e). Specifically, chronic morphine significantly reduced the soma size of NAc m.shell-projecting VTA DA neurons (*p* = 0.007, Tukey’s post-hoc test) but had no effect on NAc core-projecting neurons. There was a trend for increased size of core-projecting neurons compared to m.shell-projecting neurons in sham mice, but this did not reach statistical significance (sham m.shell: 419.7 ± 15.6, sham core: 459.3 ± 12.7, *p* = 0.08, Tukey post-hoc test). Surprisingly, within the PFC-projecting population (Fig. 2g), there was a significant increase in VTA DA soma size following chronic morphine (student t-test, t_(12)_ = 2.946, *p* = 0.012). PFC-projecting neurons were larger than NAc-projecting neurons in sham mice (PFC-sham: 544.5 ± 21.64, m.shell-sham: 419.7 ± 15.6, core-sham: 459.3 ± 12.7), consistent with differences in basal size between PBP and PN VTA DA neurons. Together, the data indicate that chronic morphine affects VTA DA soma size in a projection-specific manner: while soma size of NAc m.shell-projecting VTA DA neurons is decreased consistent with previous findings [33, 41, 43], this is not observed in VTA DA cells projecting to the NAc core. Moreover, soma size of PFC-projecting neurons is significantly increased by morphine, which is, to our knowledge, the first observation of a stimulus-induced increase in VTA DA soma size.

### Projection-specific protein expression patterns in DA neurons

**Fig. 3:**
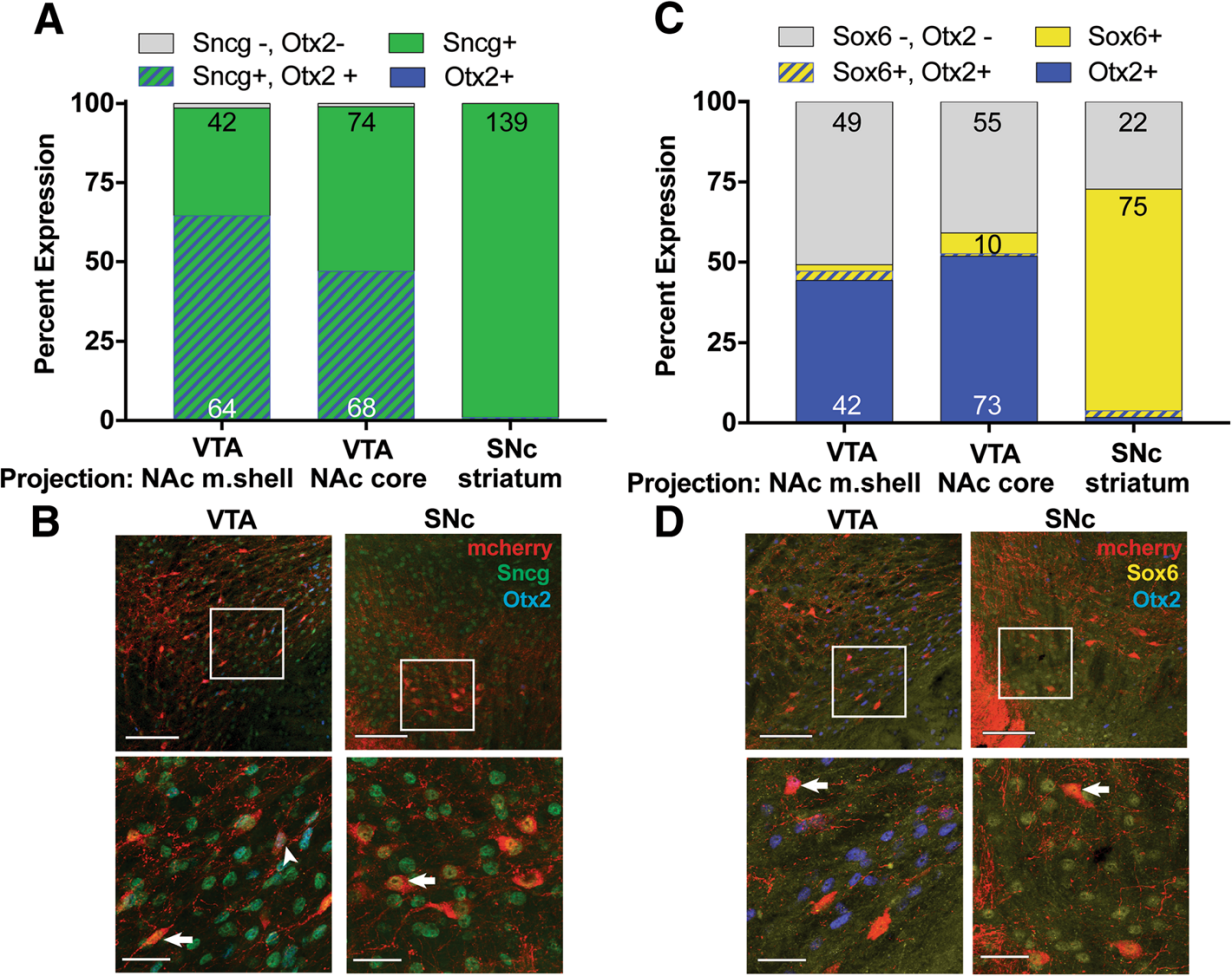
Protein markers differentiate SNc DA neurons that project to the dorsal striatum, but not VTA DA neurons that project to the NAc m.shell vs. core. **a**. Expression of Sncg (green) and Otx2 (blue) was assessed in VTA DA neurons that projected to the NAc m.shell or core or SNc DA neurons that projected the dorsal striatum (red). While ~ 50% of NAc-projecting VTA DA neurons expressed Otx2, this protein was absent from striatal-projecting SNc DA neurons. **b**. Representative images are shown of VTA (left) and SNc (right) neurons labeled with mCherry (red), Sncg (green), and Otx2 (blue). Arrowhead: Sncg+, Otx2+ cell, Arrows: Sncg+ cells. **c**. Expression of Sox6 (yellow) and Otx2 (blue) was assessed in VTA DA neurons that projected to the NAc m.shell or core or SNc DA neurons that projected the striatum (red). **d**. Representative images are shown of VTA (left) and SNc (right) neurons labeled with mCherry (red), Sox6 (yellow), and Otx2 (blue). Arrow: Otx2+ cell (VTA) or Sox6+ cell (SNc). **a**. and **c**. Data are expressed as the percent of DA projection neurons labeled, the number of neurons (for groups with > 5 neurons) is noted within the columns. **b**. and **d**. Top images were taken using a 20X objective (scale bar = 100 μm), white box illustrates image shown below (60X, scale bar = 30 μm)

Due to the surprising differences observed between VTA DA neurons projecting to NAc subregions, we next sought to determine if the NAc m.shell- and core-projecting VTA DA neurons are also molecularly distinct. DA neuron subpopulations differ transcriptionally based on single-cell RNA sequencing, including distinct gene expression patterns that distinguish striatal- and BNST-projecting neurons [40]. We therefore sought to determine if we could discriminate m.shell- from core-projecting VTA DA neurons using immunohistochemistry for two master transcriptional regulators (Otx2 and Sox6) which may differentiate SNc and VTA DA neurons [11, 15, 37] and gamma synuclein (Sncg), a protein involved in neurodevelopment, regulation of cell growth, division, and gene expression [18, 36, 46]. We assessed colocalization in VTA DA neurons labeled with AAV5-DIO-mCherry that projected to NAc m.shell and core and SNc DA neurons that projected to the striatum. Due to antibody limitations, we compared colocalization in two IHC experiments, in the first experiment cells were assessed for Sncg and Otx2 expression (Fig. 3a, b) and in the second experiment Sox6 and Otx2 were assessed (Fig. 3c, d).

While no single protein was exclusive for a single type of DA projection, there were differences in expression patterns. For example, in set 1 (Sncg and Otx2) there was a significant effect of protein marker (F_(2,30)_ = 220.1, *p* < 1.0 × 10^− 15^), a significant marker x projection interaction (F_(4,30)_ = 85.83, p < 1.0 × 10^− 15^), but no overall effect of projection (F_(2,30)_ = 2.5 × 10^− 13^, *p* > 0.99). Specifically, dorsal striatum-projecting neurons had a very different profile compared to NAc-projecting DA neurons. SNc DA neurons that projected to the dorsal striatum were almost exclusively only Sncg-positive (+) (99%, Fig. 3a) while ~ 50% of NAc-projecting VTA neurons were also Otx2+ (m.shell-projecting: 65% Sncg+ and Otx2+, core-projecting: 47% Sncg+ and Otx2+). Interestingly, across all three regions, 99% all DA neurons assessed were Sncg+, such that only 1% of DA neurons were Sncg- and Otx2-, and there were no Otx2+ only neurons identified.

In the second set of IHC, Sox6 and Otx2 co-expression was assessed (Fig. 3c, d). Using a two-way ANOVA, we determined there was a significant main effect of protein marker (F_(3,40)_ = 69.04, p < 1.0 × 10^− 15^), and protein marker x projection interaction (F_(6,40)_ = 64.94, p < 1.0 × 10^− 15^) but no overall effect of projection (F_(2,40)_ = 3.3 × 10^− 4^, p > 0.99). Overall, we found that both NAc m.shell- and core-projecting neurons in the VTA had low Sox6 expression (5 and 7%, respectively) compared to dorsal striatal-projecting SNc neurons (71% Sox6+). Together, while these data suggest that the proportions of Sncg, Otx2, and Sox6 expression can distinguish NAc-projecting VTA DA neurons from striatum-projecting SNc DA neurons, these markers do not help to molecularly define the subsets of VTA DA neurons that are differentially affected by morphine exposure.

## Discussion

Chronic opioid exposure induces lasting structural and synaptic plasticity in the mesocorticolimibic reward circuitry, particularly within VTA DA neurons. Specifically, there is an increase in VTA DA firing rate following chronic morphine treatment, and a decrease in VTA DA soma size [23, 33]. Yet VTA DA neurons are not homogeneous and recent studies that have characterized VTA DA neurons based on their projection target have noted both basal differences between subsets of VTA DA neurons as well as varied responses to rewarding and aversive stimuli. For instance, VTA DA neurons that project to the NAc l.shell are physiologically distinct from those that project to NAc m.shell or core, or to the PFC, exhibiting higher I_h_ currents, lower maximal firing rates, and reduced AMPA/NMDA ratio that are more similar to DA neurons in the SNc [24, 25]. However, despite these basal differences, cocaine increases the AMPA/NMDA ratio in VTA DA cells projecting to either the NAc m.shell or l.shell, but does not impact the AMPA-NMDA ratio of PFC-projecting VTA DA neurons [25]. Differential regulation has also been noted for aversive stimuli, as AMPA/NMDA ratio is increased in PFC-projecting VTA DA neurons, but not in NAc m.shell-projecting VTA DA neurons despite their similar baseline properties [25]. These data highlight the complexity between subsets of DA neurons, where there are differences both basally and in response to stimuli. Additionally, these differences do not neatly align, for example the basal properties of NAc l.shell-projecting VTA neurons are similar to nigrostriatal neurons, but their responses to rewarding or aversive stimuli are not.

Our findings support that morphological complexity also exists, both basally and in response to morphine exposure. Interestingly, we find that VTA DA neurons that project to the NAc m.shell exhibit the established morphine-induced decrease in soma size, while those that project mainly to the NAc core do not. This suggests results from previous studies were driven by changes in m.shell-projecting neurons. Intriguingly, we also observed a morphine-induced increase in soma size of PFC-projecting VTA DA neurons. There are multiple reasons this effect could have been missed in previous studies. For example, there was a difference in the number of cells labeled. While NAc injections robustly labeled VTA DA cells, PFC injections resulted in much more sparse labeling such that opposite regulation in this small subset of cells could have been obscured. Additionally, the VTA subregion examined likely also played a role. Previous work identifying opiate-induced decrease in soma size was primarily conducted within the PN of the VTA [32, 33, 41]. While we did observe PFC-projecting DA neurons in the PN, the majority of cells were in the PBP, suggesting these cells were likely not included in the previous analyses. Therefore, it is probable that this opposing effect was masked in previous studies due to the high density of m.shell-projecting VTA DA neurons within the region analyzed. Overall, our data support current efforts to define changes within subsets of VTA DA neurons, as these are likely defined by differences both in their projection target as well as their input [27, 48].

There is a growing body of literature supporting the importance of analyzing VTA DA neurons based on their projections to NAc subregions (m.shell, l.shell, or core) due to their distinct molecular and functional properties and behavioral responses. As noted previously, VTA DA neurons projecting to the NAc m.shell and l.shell have different basal electrophysiological properties [25]. Since we focused our efforts on the PN region of the VTA, we largely studied VTA DA neurons that projected to the NAc m.shell or core, although there were likely some l.shell-projecting neurons within the core group, consistent with previous categorization of PN VTA DA neurons [3, 24, 25]. While differences in DAT/TH and DAT/vesicular monoamine expression have been noted between m.shell- and l.shell-projecting VTA DA neurons, these markers did not distinguish between m.shell- and core-projecting VTA DA neurons in the PN subregion [24]. We therefore tested whether m.shell- and core-projecting VTA DA neurons could be identified by protein markers (Otx2, Sox6, and Sncg) implicated in distinct molecular subtypes of VTA DA neurons [40]. While no protein showed exclusive projection-specific expression, these markers were sufficient to differentiate NAc-projecting VTA DA neurons from nigrostriatal neurons. Unfortunately, they did not allow differentiation between NAc m.shell- and core-projecting VTA DA neurons. A recent study utilizing intersectional genetic approaches suggests such differentiation may be possible, as vGlut2- and Aldh1a1-Cre driver lines were found to preferentially label NAc medial shell-projecting VTA neurons [39]. Future studies to evaluate these markers, or to identify additional distinct molecular markers of VTA DA projection neurons will be crucial to allow for more detailed analysis of the functional and behavioral importance of these neurons without the necessity of cumbersome tract-tracing methods.

Importantly, NAc subregion activity may drive distinct aspects of behavioral integration of reward and aversion processing. While it is well established that dopamine release, particularly in the NAc m.shell, is important for drug reward [19, 42], there is increasing evidence that the NAc ventral and lateral shell may be involved in processing of both rewarding and aversive stimuli [1, 34, 48]. More generally, stimuli-induced increases in DA the NAc shell are thought to be important for hedonic responses, while DA release in the NAc core role is prominently involved in the acquisition and expression of motivated behavior [35], particularly in instrumental behavior tasks involving reward prediction and outcome dichotomy. Morphine exposure is associated with changes in reward behavior and induces structural and activity changes in VTA DA neurons, but it is unclear whether all VTA DA neurons respond similarly. Here we show that chronic morphine induces structural changes in discrete NAc-projecting VTA DA populations, reducing the soma size of NAc m.shell-projecting VTA DA neurons while having no effect on nearby core/l.shell-projecting neurons. Decreased VTA DA soma size is correlated with increased DA neuronal activity, and decreasing firing rate via overexpression of a potassium channel is sufficient to normalize soma size changes induced by morphine or Clock mutation [13, 33]. Thus, our data may suggest that opiates differentially affect the activity of subsets of VTA DA neurons. This hypothesis deserves to be explicitly tested in future studies, preferably linking soma size and activity in individual neurons. Overall, our results suggest that behaviors associated with morphine-induced changes in morphology such as reward tolerance [41] may be driven by distinct VTA subcircuits.

Importantly, recent work on NAc m.shell and l.shell highlights that projection-specific subsets of VTA DA neurons also receive distinct NAc subregion GABAergic input [48]. It is well established that chronic morphine increases VTA DA neuronal activity [20, 23, 33]. But whether morphine-induced changes in GABAergic tone [17, 20] or excitatory regulation [8, 30] differentially affect subsets of VTA DA neurons remains unclear. Taken together, these data make it clear that it will be necessary to address whether specific forms of input drive projection-specific changes in VTA DA neuron structure and activation induced by morphine in future studies.

In addition to the differences in NAc-projecting VTA DA neurons, we also determined that the structure of PFC-projecting VTA DA neurons was altered by chronic morphine exposure, as soma size was increased. While multiple stimuli in addition to opiates have been found to decrease DA soma size (sexual experience, Clock gene mutation [13, 38]), to our knowledge, this is the first observation of a stimulus-induced increase in VTA DA soma size. However, given that this effect was not evident in previous opiate studies likely due to the sparse labeling of these cells compared to NAc-projecting neurons, or the VTA subregion analyzed, it is possible that known stimuli could also produce a similar bidirectional effect on VTA DA soma size. While there are practical challenges to analyzing all projection target region x VTA subregion combinations given unequal cell distributions (e.g. number of PFC-projecting VTA DA cells in PN is much less than the number of NAc medial shell-projecting neurons), a more comprehensive definition of the VTA DA neurons under study will be important going forward. Opposing effects of chronic opioids on morphological adaptations across subsets of VTA DA neurons has been observed previously. An electron microscopy study found that chronic morphine increased the diameter of dendrites on VTA DA neurons in the PBP, but decreased the diameter of dendrites on VTA DA neurons in the PN [29]. Critically, these studies used retrograde tracers and found that PFC-projecting neurons were primarily found in the PBP while NAc-projecting neurons were more prominently labeled in the PN. These results are consistent with the current data, where we observe a morphine-induced increase in PFC-projecting neurons (in PBP) and decrease in NAc m.shell-projecting neurons (in PN). Future studies verifying dendritic changes in PFC- vs. NAc-projecting DA neurons as well as studies of morphine-induced changes in synaptic plasticity will allow the development of a more refined model of opiate-induced changes in mesocorticolimbic circuit activity. It will also be necessary to establish whether projection-specific changes in VTA DA morphology changes contribute to the behavioral changes associated with chronic opiate administration, such as reward tolerance [33, 41]. Overall, the current results demonstrate that VTA DA neurons differ in soma size by both subregion and projection, and that these differences extend to their morphological responses to chronic opiate exposure. Moreover, our work supports the necessity of studying opiate-induced adaptations in VTA subcircuits, as changes are likely mediated by discrete sets of neurons, with opiate adaptations possibly distinct from other stimuli [32].

## Additional files

Additional file 1: **Table S1.** Soma size data from individual mice used in studies. (TIF 29715 kb)

Additional file 2: **Table S2.** Soma size data of all individual cells used in studies. (XLSX 63 kb)

## References

[1] Al-Hasani R, McCall JG, Shin G, Gomez AM, Schmitz GP, Bernardi JM, Pyo CO, Park SI, Marcinkiewcz CM, Crowley NA, Krashes MJ, Lowell BB, Kash TL, Rogers JA, Bruchas MR (2015). Distinct subpopulations of nucleus Accumbens Dynorphin neurons drive aversion and reward. Neuron.

[2] Barrot M (2014). The ventral tegmentum and dopamine: a new wave of diversity. Neuroscience.

[3] Beier KT, Steinberg EE, DeLoach KE, Xie S, Miyamichi K, Schwarz L, Gao XJ, Kremer EJ, Malenka RC, Luo L (2015). Circuit architecture of VTA dopamine neurons revealed by systematic input-output mapping. Cell.

[4] Boscarino JA, Rukstalis M, Hoffman SN, Han JJ, Erlich PM, Gerhard GS, Stewart WF (2010). Risk factors for drug dependence among out-patients on opioid therapy in a large US health-care system. Addiction.

[5] Centers for Disease Control and Prevention (2017) Opioid Overdose: Overview of an epidemic. In. Available at: https://www.cdc.gov/drugoverdose/data/index.html. Accessed 14 May 2018.

[6] Cetin A, Komai S, Eliava M, Seeburg PH, Osten P (2006). Stereotaxic gene delivery in the rodent brain. Nat Protoc.

[7] Chaudhury D (2013). Rapid regulation of depression-related behaviours by control of midbrain dopamine neurons. Nature.

[8] Chen M, Zhao Y, Yang H, Luan W, Song J, Cui D, Dong Y, Lai B, Ma L, Zheng P (2015). Morphine disinhibits glutamatergic input to VTA dopamine neurons and promotes dopamine neuron excitation. Elife.

[9] Chu NN, Zuo YF, Meng L, Lee DY, Han JS, Cui CL (2007). Peripheral electrical stimulation reversed the cell size reduction and increased BDNF level in the ventral tegmental area in chronic morphine-treated rats. Brain Res.

[10] Chung AS, Miller SM, Sun Y, Xu X, Zweifel LS (2017). Sexual congruency in the connectome and translatome of VTA dopamine neurons. Sci Rep.

[11] Chung CY, Licznerski P, Alavian KN, Simeone A, Lin Z, Martin E, Vance J, Isacson O (2010). The transcription factor orthodenticle homeobox 2 influences axonal projections and vulnerability of midbrain dopaminergic neurons. Brain.

[12] Cooper S, Robison AJ, Mazei-Robison MS (2017). Reward circuitry in addiction. Neurotherapeutics.

[13] Coque L, Mukherjee S, Cao JL, Spencer S, Marvin M, Falcon E, Sidor MM, Birnbaum SG, Graham A, Neve RL, Gordon E, Ozburn AR, Goldberg MS, Han MH, Cooper DC, McClung CA (2011). Specific role of VTA dopamine neuronal firing rates and morphology in the reversal of anxiety-related, but not depression-related behavior in the ClockDelta19 mouse model of mania. Neuropsychopharmacology.

[14] Darnall BD, Stacey BR, Chou R (2012). Medical and psychological risks and consequences of long-term opioid therapy in women. Pain Med.

[15] Di Salvio M, Di Giovannantonio LG, Omodei D, Acampora D, Simeone A (2010). Otx2 expression is restricted to dopaminergic neurons of the ventral tegmental area in the adult brain. Int J Dev Biol.

[16] Fischer SJ, Arguello AA, Charlton JJ, Fuller DC, Zachariou V, Eisch AJ (2008). Morphine blood levels, dependence, and regulation of hippocampal subgranular zone proliferation rely on administration paradigm. Neuroscience.

[17] Ford CP, Mark GP, Williams JT (2006). Properties and opioid inhibition of mesolimbic dopamine neurons vary according to target location. J Neurosci.

[18] Galvin JE, Schuck TM, Lee VM, Trojanowski JQ (2001). Differential expression and distribution of alpha-, beta-, and gamma-synuclein in the developing human substantia nigra. Exp Neurol.

[19] Ikemoto S (2007). Dopamine reward circuitry: two projection systems from the ventral midbrain to the nucleus accumbens-olfactory tubercle complex. Brain Res Rev.

[20] Johnson SW, North RA (1992). Opioids excite dopamine neurons by hyperpolarization of local interneurons. J Neurosci.

[21] Juarez B, Han MH (2016). Diversity of dopaminergic neural circuits in response to drug exposure. Neuropsychopharmacology.

[22] Juarez B (2017). Midbrain circuit regulation of individual alcohol drinking behaviors in mice. Nat Commun.

[23] Koo JW, Mazei-Robison MS, Chaudhury D, Juarez B, LaPlant Q, Ferguson D, Feng J, Sun H, Scobie KN, Damez-Werno D, Crumiller M, Ohnishi YN, Ohnishi YH, Mouzon E, Dietz DM, Lobo MK, Neve RL, Russo SJ, Han MH, Nestler EJ (2012). BDNF is a negative modulator of morphine action. Science.

[24] Lammel S, Hetzel A, Hackel O, Jones I, Liss B, Roeper J (2008). Unique properties of mesoprefrontal neurons within a dual mesocorticolimbic dopamine system. Neuron.

[25] Lammel S, Ion DI, Roeper J, Malenka RC (2011). Projection-specific modulation of dopamine neuron synapses by aversive and rewarding stimuli. Neuron.

[26] Lammel S, Lim BK, Malenka RC (2014). Reward and aversion in a heterogeneous midbrain dopamine system. Neuropharmacology.

[27] Lammel S, Lim BK, Ran C, Huang KW, Betley MJ, Tye KM, Deisseroth K, Malenka RC (2012). Input-specific control of reward and aversion in the ventral tegmental area. Nature.

[28] Lammel S, Steinberg EE, Foldy C, Wall NR, Beier K, Luo L, Malenka RC (2015). Diversity of transgenic mouse models for selective targeting of midbrain dopamine neurons. Neuron.

[29] Lane DA, Lessard AA, Chan J, Colago EE, Zhou Y, Schlussman SD, Kreek MJ, Pickel VM (2008). Region-specific changes in the subcellular distribution of AMPA receptor GluR1 subunit in the rat ventral tegmental area after acute or chronic morphine administration. J Neurosci.

[30] Margolis EB, Hjelmstad GO, Fujita W, Fields HL (2014). Direct bidirectional mu-opioid control of midbrain dopamine neurons. J Neurosci.

[31] Margolis EB, Lock H, Hjelmstad GO, Fields HL (2006). The ventral tegmental area revisited: is there an electrophysiological marker for dopaminergic neurons?. J Physiol.

[32] Mazei-Robison MS, Appasani R, Edwards S, Wee S, Taylor SR, Picciotto MR, Koob GF, Nestler EJ (2014). Self-administration of ethanol, cocaine, or nicotine does not decrease the soma size of ventral tegmental area dopamine neurons. PLoS One.

[33] Mazei-Robison MS (2011). Role for mTOR signaling and neuronal activity in morphine-induced adaptations in ventral tegmental area dopamine neurons. Neuron.

[34] McCutcheon JE, Ebner SR, Loriaux AL, Roitman MF (2012). Encoding of aversion by dopamine and the nucleus accumbens. Front Neurosci.

[35] Namburi P, Al-Hasani R, Calhoon GG, Bruchas MR, Tye KM (2016). Architectural representation of valence in the limbic system. Neuropsychopharmacology.

[36] Pan ZZ, Bruening W, Giasson BI, Lee VM, Godwin AK (2002). Gamma-synuclein promotes cancer cell survival and inhibits stress- and chemotherapy drug-induced apoptosis by modulating MAPK pathways. J Biol Chem.

[37] Panman L, Papathanou M, Laguna A, Oosterveen T, Volakakis N, Acampora D, Kurtsdotter I, Yoshitake T, Kehr J, Joodmardi E, Muhr J, Simeone A, Ericson J, Perlmann T (2014). Sox6 and Otx2 control the specification of substantia nigra and ventral tegmental area dopamine neurons. Cell Rep.

[38] Pitchers KK, Coppens CM, Beloate LN, Fuller J, Van S, Frohmader KS, Laviolette SR, Lehman MN, Coolen LM (2014). Endogenous opioid-induced neuroplasticity of dopaminergic neurons in the ventral tegmental area influences natural and opiate reward. J Neurosci.

[39] Poulin JF, Caronia G, Hofer C, Cui Q, Helm B, Ramakrishnan C, Chan CS, Dombeck DA, Deisseroth K, Awatramani R (2018). Mapping projections of molecularly defined dopamine neuron subtypes using intersectional genetic approaches. Nat Neurosci.

[40] Poulin JF, Zou J, Drouin-Ouellet J, Kim KY, Cicchetti F, Awatramani RB (2014). Defining midbrain dopaminergic neuron diversity by single-cell gene expression profiling. Cell Rep.

[41] Russo SJ, Bolanos CA, Theobald DE, DeCarolis NA, Renthal W, Kumar A, Winstanley CA, Renthal NE, Wiley MD, Self DW, Russell DS, Neve RL, Eisch AJ, Nestler EJ (2007). IRS2-Akt pathway in midbrain dopamine neurons regulates behavioral and cellular responses to opiates. Nat Neurosci.

[42] Sesack SR, Grace AA (2010). Cortico-basal ganglia reward network: microcircuitry. Neuropsychopharmacology.

[43] Sklair-Tavron L, Shi WX, Lane SB, Harris HW, Bunney BS, Nestler EJ (1996). Chronic morphine induces visible changes in the morphology of mesolimbic dopamine neurons. Proc Natl Acad Sci U S A.

[44] Stamatakis AM, Jennings JH, Ung RL, Blair GA, Weinberg RJ, Neve RL, Boyce F, Mattis J, Ramakrishnan C, Deisseroth K, Stuber GD (2013). A unique population of ventral tegmental area neurons inhibits the lateral habenula to promote reward. Neuron.

[45] Stuber GD, Stamatakis AM, Kantak PA (2015). Considerations when using cre-driver rodent lines for studying ventral tegmental area circuitry. Neuron.

[46] Surguchev AA, Surguchov A (2017). Synucleins and gene expression: ramblers in a crowd or cops regulating traffic?. Front Mol Neurosci.

[47] Witten IB, Steinberg EE, Lee SY, Davidson TJ, Zalocusky KA, Brodsky M, Yizhar O, Cho SL, Gong S, Ramakrishnan C, Stuber GD, Tye KM, Janak PH, Deisseroth K (2011). Recombinase-driver rat lines: tools, techniques, and optogenetic application to dopamine-mediated reinforcement. Neuron.

[48] Yang H, de Jong JW, Tak Y, Peck J, Bateup HS, Lammel S (2018). Nucleus Accumbens subnuclei regulate motivated behavior via direct inhibition and disinhibition of VTA dopamine subpopulations. Neuron.

