# Supplementary figures and images for: Determination of circuit-specific morphological adaptations in ventral tegmental area dopamine neurons by chronic morphine
Source: Mol Brain. 2019 Feb 8;12:10. doi: 10.1186/s13041-019-0435-6 (PMC6368752; doi:10.1186/s13041-019-0435-6)

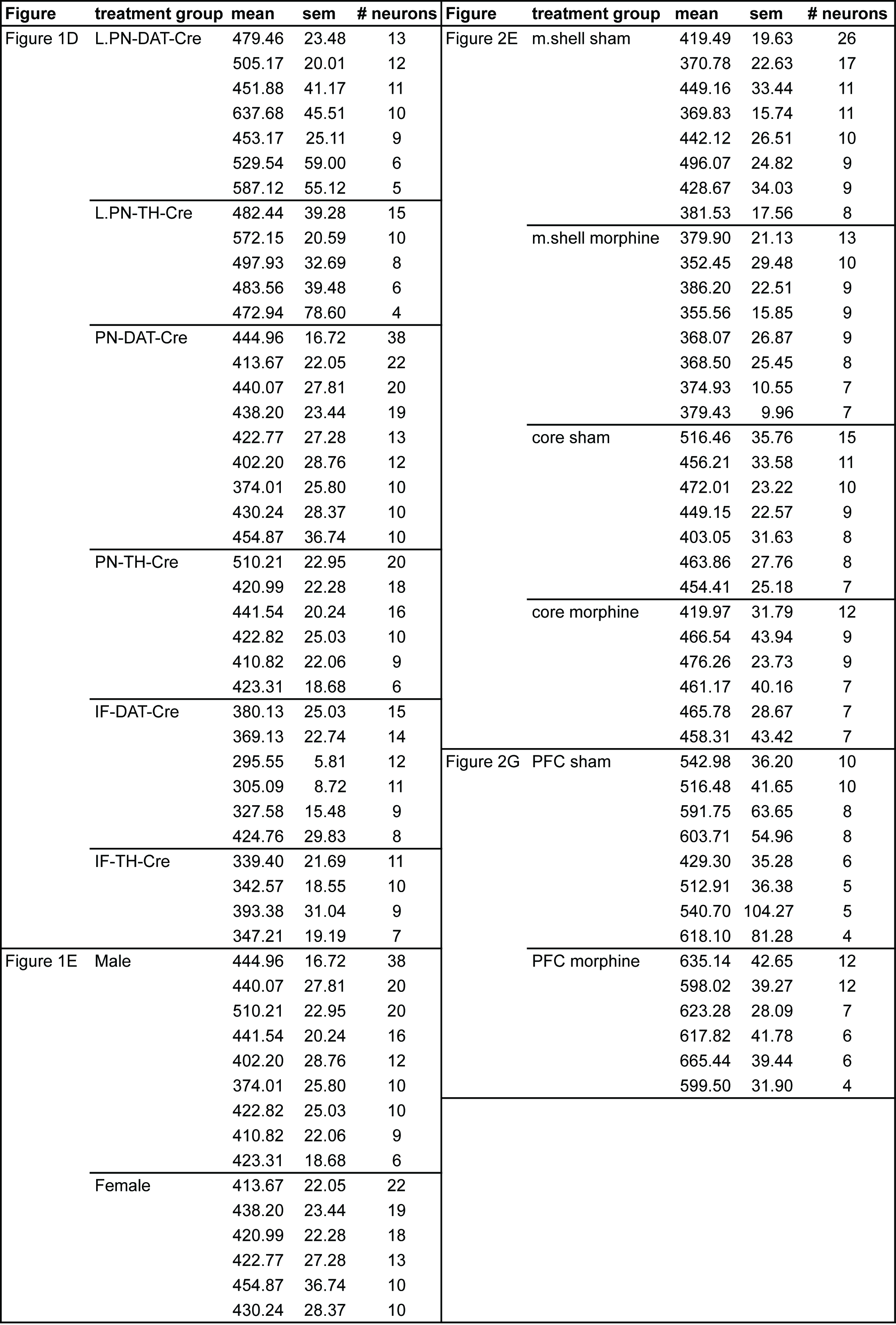

Supplement: Supplementary file 1 — Table S1. Soma size data from individual mice used in studies. (TIF 29715 kb) [file 13041_2019_435_MOESM1_ESM.tif]
